# The role of the adaptive immune system and T cell dysfunction in neurodegenerative diseases

**DOI:** 10.1186/s12974-022-02605-9

**Published:** 2022-10-08

**Authors:** Alexa DeMaio, Shikhar Mehrotra, Kumar Sambamurti, Shahid Husain

**Affiliations:** 1 Department of Ophthalmology, Storm Eye Institute, Room 713, Medical University of South Carolina, 167 Ashley Ave, SC 29425 Charleston, USA; 2 Department of Surgery, Hollings Cancer Center, Medical University of South Carolina, SC 29425 Charleston, USA; 3 Department of Neuroscience, Medical University of South Carolina, SC 29425 Charleston, USA

**Keywords:** T cells, Neurodegeneration, Inflammation, Immune system, Lymphocytes

## Abstract

The adaptive immune system and associated inflammation are vital in surveillance and host protection against internal and external threats, but can secondarily damage host tissues. The central nervous system is immune-privileged and largely protected from the circulating inflammatory pathways. However, T cell involvement and the disruption of the blood–brain barriers have been linked to several neurodegenerative diseases including Parkinson's disease, Alzheimer’s disease, and multiple sclerosis. Under normal physiological conditions, regulatory T cells (Treg cells) dampen the inflammatory response of effector T cells. In the pathological states of many neurodegenerative disorders, the ability of Treg cells to mitigate inflammation is reduced, and a pro-inflammatory environment persists. This perspective review provides current knowledge on the roles of T cell subsets (e.g., effector T cells, Treg cells) in neurodegenerative and ocular diseases, including uveitis, diabetic retinopathy, age-related macular degeneration, and glaucoma. Many neurodegenerative and ocular diseases have been linked to immune dysregulation, but the cellular events and molecular mechanisms involved in such processes remain largely unknown. Moreover, the role of T cells in ocular pathologies remains poorly defined and limited literature is available in this area of research. Adoptive transfer of Treg cells appears to be a vital immunological approach to control ocular pathologies. Similarities in T cell dysfunction seen among non-ocular neurodegenerative diseases suggest that this area of research has a great potential to develop better therapeutic agents for ocular diseases and warrants further studies. Overall, this perspective review article provides significant information on the roles of T cells in numerous ocular and non-ocular neurodegenerative diseases.

## Adaptive immune responses and pathological conditions

The immune system can be divided into innate and adaptive immune responses and is designed to protect the body from disease-causing pathogens. The innate immune system is the body's first line of defense and requires no lag time to mount a response to infection. Innate immunity is largely nonspecific. Adaptive immunity is much more specific and provides long-lasting protection against pathogens [5, 176]. The cells that carry out adaptive immune responses are called lymphocytes and are classified into B lymphocytes and T lymphocytes, which accomplish humoral and cell-mediated immune responses, respectively [279]. In the humoral immune response, a B cell receptor reacts with a specific antigen stimulating the cell to synthesize and secrete antibodies, also known as immunoglobulins. These antibodies play crucial roles in neutralizing pathogens [5, 289]. Antibodies recognize epitopes on a single specific molecule (e.g., protein or carbohydrate) called an antigen. When an antigen, such as a virus or microbial toxin (e.g., tetanus or diphtheria toxin), binds an antibody, it can no longer bind to receptors on host cells and is therefore neutralized [81]. T lymphocytes (T cells) arise in the bone marrow and mature in the thymus. They are classified into two major categories CD8 + and CD4 + cells based on their effector functions and recognition of different classes of MHC molecules. CD8 + cells defend the host against intracellular pathogens such as viruses and cancer, as they can detect surface antigens displayed by infected cells [152–154]. Cytotoxic CD8 + cells do not recognize free antigens but bind short peptide antigens expressed on the major histocompatibility complex (MHC) class I protein on the surface of most cells [131, 152–154]. MHC class I molecules are found on all nucleated cells and are important in T cell activity against viruses, but MHC class II molecules are only found on APCs, which help with the proliferation of B cells [152–154]. An antigen-presenting cell (APC; e.g., dendritic cells, macrophages, Langerhans cells, and B cells) must stimulate a cytotoxic T cell to activate it. A pathogen-activated APC will subsequently travel to secondary lymphoid tissues, such as lymph nodes [178]. After activation by an APC, the T cell will expand its population to eliminate the specific pathogen [152–154]. Once a population of T cells has been created to combat pathogens, memory T cells will provide lifelong immunity against the pathogen [91]. Mechanistically, CD8 + T cells bind to MHC class I, whereas CD4 + T cells bind to MHC class II [110]. Once a CD8 + T cell has been primed by an APC cell, it is activated and ready to kill infected or invading cells. When the CD8 + T cell binds to its target antigen on the surface of an infected cell, it will release lytic granules containing perforins and granzymes that create pores in the target cell membrane or it induces apoptosis via Fas ligand and caspase activation [362]. In addition, CD8 + T cells release cytokines including IFN-γ, TNF-α, and TNF-β. These cytokines will further inhibit viral replication, activate macrophages, and upregulate MHC class I expression. MHC class I expression increases the detection of an infected cell, as T cells cannot recognize cells without MHC molecules [121, 152–154].

CD4 + T cells were originally subdivided into two groups based on their effector functions: Th1 and Th2 cells. These Th1 and Th2 cells are antagonistic and maintain a balance under physiological conditions. Th1 cells, generally, secrete pro-inflammatory molecules such as IFN-γ, TNF-α, TNF-β, and IL-1β; while Th2 cells counteract inflammation with IL-4, IL-5, IL-6, IL-10, and IL-13 [25, 233, 361]. Until the mid-1990s, scientists were not aware of other subsets of CD4 + cells [63], but now CD4 + T cells have been expanded further into Th9, Th17, Th22, Treg cells, and T follicular cells [9, 110]. Th9, Th17, and Th22 T cell subsets secrete different pro-inflammatory cytokines and have been implicated in autoimmune and inflammatory diseases [193, 311, 328]. For example, in allergic asthma Th2, Th9, and Th17 cells play a role in the pathogenesis of the disease through cytokine secretion and the activation of mast cells and eosinophils, which leads to airway hyperactivity [174]. While the ability of T cells to cause inflammation may be necessary for proper immune function, a mechanism to regulate this response is also required. T regulatory (Treg) cells have been shown to play key roles in their regulation [14, 180, 244, 267, 342]. As evident from the name, Treg cells regulate and suppress the potentially dangerous effects of T cells and ultimately promote tolerance. Numerous functions of Treg cells have been documented including suppression of asthma and allergy [45, 51, 287], induction of tolerance to dietary antigens [12, 106], protection of commensal bacteria from elimination by the immune system [40], promotion of maternal–fetal tolerance [135], and prevention of autoimmune disease [63, 117, 333, 334]. The role of Treg cells is not fully understood, and it remains a key area of research, specifically in ocular pathology. However, it has become increasingly clear that Treg cells are required to maintain homeostasis. As referenced above, a hyperactive inflammatory immune response can harm host cells. Conversely, a lack of a properly functioning immune system can lead to life-threatening infections, so a balance must be struck between the two states. Treg cells maintain this balance by playing a suppressive role and preventing a pro-inflammatory response [189]. Numerous disease conditions such as systemic sclerosis [215], gestational diabetes [286], atherosclerosis [138], COPD [149], among many others have reported T cell subsets imbalance as a causative factor in disease pathogenesis.

## Tolerance and autoimmunity

The immune system is designed to target foreign pathogens and leave host cells unharmed via a process known as tolerance. Autoimmunity occurs when a host loses self-tolerance. In other words, autoimmunity is a process of an immune response against the host leading to self-cell damage. Different autoimmune conditions can target specific areas of the body like pemphigus vulgaris and pemphigoid, which targets the skin [122], or systemic lupus erythematosus which is disseminated and targets the entire body [182]. An important aspect of autoimmunity is the presence of autoreactive lymphocytes. Auto-reactivity can be triggered through molecular mimicry, which is immunological cross-reactivity between host and foreign antigens. In this condition, foreign antigens closely resemble host antigens resulting in an erroneous self-directed attack on host tissue [67]. Autoreactive lymphocytes can also develop during the initial lymphocyte creation. When lymphocytes undergo genetic rearrangement, some are reactive against self-antigens, but under homeostatic conditions these lymphocytes undergo thymic deletion and undergo apoptosis [331]. Although up to 98% of T cells do not make it through the selection process [24], some autoreactive lymphocytes will escape the process of elimination even in healthy individuals [266]. One of the functions of Treg cells is to prevent the activation of these autoreactive lymphocytes and therefore protect the body from self-attack [297]. FoxP3 knockdown, a marker for Treg cells, leads to a loss of functional Treg cells in Scurfy mice [231]. The lack of Treg cells leads to a fatal autoimmune response by 3–4 weeks of age, demonstrating their critical role in immune tolerance. T cell activation suppression via the induction of anergy, a long-term state of T cell hypo-responsiveness, is the second mechanism of its modulation [43]. Inducing anergy will prevent T cells from undergoing clonal proliferation, providing another potential fail-safe against autoimmunity. Recent studies have shown a strong connection between Treg cells and anergy, anergic T cells can convert into Treg cells, suggesting that persistent anergic T cells may serve as a reservoir for Treg cells, though the mechanism remains largely unknown [246, 292].

Autoimmune diseases affect millions of people with increasing frequency in developing countries, making them an important area of study [265]. Some autoimmune conditions are hereditary, and many primarily affect women, but they typically require a trigger or underlying susceptibility [62]. Autoimmunity is multifactorial and involves genetic, environmental, hormonal, immunological, and unknown triggers [302]. Modifiable risk factors for autoimmune diseases include infection [185], vaccination [329], drugs [72], smoking [268], UV light exposure [166] and obesity [302]. Studies have also shown that people with autoimmune diseases are less active than the rest of the population, suggesting physical activity may play a regulatory role in autoimmunity [284].

## Adaptive immunity and Alzheimer’s disease

Alzheimer’s disease (AD) is a neurodegenerative disorder and one of the most common causes of senile dementia worldwide. The loss of memory and cognitive decline was seen in AD is progressive, irreversible, and is usually seen in the elderly population. Characteristic features of the disease are the accumulation of β-amyloid plaque deposits as well as neurofibrillary tangles of hyper-phosphorylated tau in the brain [30, 78, 82, 339]. However, the familial AD (FAD) mouse models combined with frontotemporal dementia-linked human microtubule associated tau fail to show the extensive and progressive neurodegeneration reported in human patients [230]. Therefore, there is a major gap in our understanding of the steps leading to neurodegeneration and irreversible dementia. The evidence for immune system involvement in AD is compelling. Several studies have implicated both microglia and the innate immune system in the neurodegeneration seen in AD [29, 280]. Studies have also shown the involvement of the adaptive immune system in the pathology of AD. T cells have been identified in the brain parenchyma of postmortem Alzheimer’s patients [321] and T cell abnormalities have also been identified in the blood and cerebral spinal fluid of Alzheimer’s patients [104, 212]. Studies in a mouse model have also shown that β-amyloid promotes T cell infiltration, and it interferes with proper T cell functioning, including activation and antigen presentation, suggesting that failure to mount a protective immune response may contribute to AD pathology [95]. A breakdown in antigen presentation may contribute to an immune inability to clear β-amyloid. Alterations in the peripheral lymphocyte profiles have also been demonstrated in Alzheimer’s disease [49, 50, 184, 262]. Recently, studies have also shown an association between higher levels of CD 4 + cell counts and an increased risk of AD [90]. Most research has focused on T cells in AD, but a few recent findings have also highlighted the roles of B cells in the disease. One study suggests that B cell depletion may prevent disease progression, which is an interesting theory in light of recent identification of resident B cells in the dura matter [171, 275]. This data suggests that alterations in B lymphocyte number, subsets, and production of autoantibodies may all be involved in AD pathology and progression of the disease [33, 270, 271, 298].

Recent studies focusing on cerebrospinal fluid (CSF) biomarkers have consistently disregarded the cellular infiltrates, treating them as artifacts of collection. However, a careful examination of CSF by cutting-edge methods of mass cytometry, revealed a consistent increase in CD8^+^ T effector memory CD45RA^+^ (T_EMRA_) cells, and their association with cognitive impairment [104]. Additionally, single-cell RNA sequencing and artificial intelligence revealed an increase in T cell receptor (TCR) signaling, and further analysis identified clonally expanded CD8^+^ T_EMRA_ cells targeting Epstein–Barr viral antigens. These studies show that T cells in the CSF play the same role as peripheral T cells in surveillance and maintenance of the intrathecal space. The existence of immune plasticity can be both beneficial and detrimental, depending on the target, extent of activity, and resolution of the inflammatory cascade [172, 278]. This complex pathway is consistent with findings showing that depletion of T cells prevented hippocampal infiltration and spatial memory deficits in AD models [186]. Acute inflammatory responses may be neuroprotective and promote plaque clearance, but chronic inflammatory responses may be detrimental due to excessive collateral tissue damage [4, 273]. Therefore, lymphocyte physiology is altered during AD pathogenesis and these changes likely play a role in the progression of the disease.

Immunotherapy is an area of high interest in AD. The recent approval of a monoclonal antibody called aducanumab that selectively targets aggregated forms of amyloid β in AD patients may help in preventing early disease progression [94, 277, 282]. This antibody is produced using selectively reactive B cells, and shows that the adaptive immune system may hold the key for better AD treatments and prognoses. However, the approval of aducanumab has been met with controversy and many questions arose about the efficacy of this drug [173, 282]. While the clinical success of aducanumab remains in question, immunotherapy using other antibodies will continue to be the focus of research for future drug development in this field. Overall, research involving monoclonal antibodies that recruit T cells (e.g., effector T cells and Tregs) to target amyloid β may hold promise in restoring the disrupted immune balance in AD.

## Adaptive immunity and Parkinson’s disease

Parkinson’s disease (PD) is another example of an inflammatory disease process causing progressive neurodegeneration of the dopaminergic neurons in the substantia nigra of the brain [20]. Aggregates of misfolded α-synuclein (α-syn protein accumulate in the brain of PD patients, similar to the protein aggregates observed in Alzheimer’s disease. Patients suffering from PD experience motor symptoms such as tremors, muscle rigidity, and slowness of movement (bradykinesia but they can also experience cognitive impairment. The involvement of immune cells in PD has been suggested. For example, studies have shown activated microglia in the substantia nigra of postmortem Parkinson’s disease patients [207] and potential role of the adaptive immune system in PD has been shown [132, 163, 250].

Parkinson’s disease is considered to be a systemic inflammatory disorder because elevated pro-inflammatory cytokines are found in the blood of PD patients [27, 79, 103, 140, 257, 259, 304]. An increased level of inflammatory cytokines is believed to be due to T cell activation. Studies have shown that activation of T cells in response to Parkinsonian α-synuclein peptides and the inflammation observed in PD could be, partly, due to the involvement of autoreactive T cells [23, 307]. Autoreactive T cells are central to autoimmune pathology, so their presence may suggest the disease is autoimmune in nature. Autoimmune conditions are also characterized by the creation of autoantibodies by B cells against self-antigens which has been demonstrated in the peripheral blood of PD patients [83, 235, 347]. Recently, a study has shown an alteration in B cells population, which may play a role in PD [332]. Additionally, studies have also shown abnormal profiles of B cells and T follicular cells, indicating a polarization towards an inflammatory phenotype [195].

Regardless of the autoimmune nature of Parkinson’s disease, T cells, and the adaptive immune system are believed to contribute to the disease development [132, 163, 250]. Several studies reported a Th1 bias in PD patients and experimental animal models [15, 53, 181]. As Th1 cells are pro-inflammatory, a shift favoring their expansion is congruent with the theory that neuroinflammation plays a role in PD. Another study has shown that CD4 + cells were the primary mediator of dopaminergic damage [31], while other studies have shown a decrease in the circulating CD4 + cell population in PD [18, 301]. If CD4 + T cells are the main mediators of disease, decreased circulating levels seem contradictory. However, the overall decrease in CD4 + T cells may be attributed to a decrease in Th17, Th2, and Treg cells and not the Th1 lineage [181]. Maintenance of the Th1 lineage despite a decrease in the other subsets would still fit with the studies citing a Th1 bias, though not all studies are in agreement [229]. One study marked an observed increase in the proportional CD4 + and CD3 + T cells as well as the CD4 + /CD8 + ratio in PD patients [52]; whereas, another study showed a decrease in the CD3 + , CD8 + T cells and B lymphocyte subsets in addition to a decrease in CD4 + T cells [114, 229]. Reasons for a decrease in the CD4 + T cell population may be explained by a study done by Calopa et al. which found increased susceptibility to apoptosis in the CD4 + T cells in the peripheral blood of PD patients [34]. Other conflicting data exist on the Th17 subset. While Kustrimovic et al. found that Th17 cells were decreased in the blood of PD patients, other studies have found that peripheral Th17 cells were increased in PD patients [49, 50, 53, 349]. There is conflicting evidence on the relative prevalence of each subset level, but overall, many abnormalities in T cells subsets populations have been reported in PD in favor of an inflammatory phenotype. More research is needed to clarify how effector T cell populations are affected in PD.

Treg cells likely play an opposing role in PD to the inflammatory T cell subsets by suppressing their effector functions and preventing rampant inflammation [139]. This hypothesis is supported by studies that showed that the transfer of Treg cells could provide neuroprotection in mouse models of PD [260, 261]. The beneficial nature of Treg cells in PD may explain why global T cell deficiency worsened the motor deficits seen in a Parkinson mouse model by decreasing effector T cells and inadvertently reducing the protective effects of Treg cells [340]. However, as PD is plagued by neuroinflammation, it is evident that Treg cells cannot properly execute their job for unknown reasons, and this dysfunction may contribute to disease progression [165]. The idea that Treg cells are unable to adequately function is supported by a study that has shown an impaired ability of Treg cells to suppress effector T cells in PD [274]. This breakdown of functioning furthers the theory that an inflammatory imbalance is observed in PD. In spite of discrepancies relating to T cell subset numbers, a change in the ability of Treg cells to function properly would result in homeostatic deviations regardless of cell numbers. More research is needed to clarify the uncertainties in the field, but most of the literature available suggests that the inflammation observed during PD is partly due to a T cell subset imbalance, which favors inflammation [52, 196]. Better understanding of the mechanisms behind PD and how the immune system is involved will hopefully lend to the development of effective therapies for PD.

## Adaptive immunity and multiple sclerosis

Multiple sclerosis (MS) is another neurodegenerative disease of the central nervous system that causes motor and sensory deficits [245]. The main hallmark of the disease is the presence of disseminated focal lesions or plaques in the CNS where demyelination and gliosis occur with relative axonal sparing [247]. Multiple sclerosis is an inflammatory disease similar to other neurodegenerative conditions, with macrophages and microglia contributing to the pathology. Other peripheral immune cells are also likely to be involved in the demyelination, including T cells (CD4 + and CD8 +), B lymphocytes, plasma cells, and dendritic cells [75, 129], and interactions between macrophages and lymphocytes may be part of the underlying pathogenesis [58].

Autoreactive T cells are an important part of autoimmune pathology in MS. Due to the extensive involvement of lymphocytes in MS plaques, the question has been raised as to whether the cause of the inflammation observed in MS may be autoimmune-dependent. A widely used animal model of MS, known as experimental autoimmune encephalomyelitis (EAE), is largely CD4 + T cell-driven [60]. Moreover, data have shown a link between CD4 + cytotoxic lymphocytes, disease severity, and plaque activity in MS patients [102, 241]. Despite haziness surrounding the underlying cause of the inflammation seen in MS, the evidence of T cell involvement is strong. It has been shown that activated T cells can induce experimental autoimmune encephalomyelitis in healthy mice [97, 177, 209] and that global reduction of most lymphocytes via alemtuzumab can improve MS pathology [161, 249]. Th1 cells have been implicated as important detrimental players in MS due to their ability to stimulate M1 macrophages through the secretion of TNF‐α and IFN‐γ, which are important mediators of inflammation and cellular damage [192]. Most of the earlier research in MS focused on the role of T cells, however, recently researchers have also acknowledged a cooperation between B cells and T cells in MS pathogenesis [155]. For example, B cells have been shown to work in tandem with T cells in human MS pathology [155]. Though more research is needed on the interplay between T cells and B cells in multiple sclerosis, a similar shift towards a pro-inflammatory B cell state has been suggested [85, 86, 193, 194]. The mechanisms involved in B cell-induced pathology in MS remains largely unknown. As of 2019, all approved MS disease-modifying therapies impact B cells in some way, such as depletion of CD20 + B cells [270]. However, not all B cell targeted therapies in MS have created positive results. One clinical trial using an experimental B cell depleting therapy for MS was terminated early due to worsening of disease progression [164]. This suggests that the role of B cells in MS is complex, and more research is needed to better understand the exact roles of B cells in MS pathology.

Unlike the harmful effects of the inflammatory T cell subsets discussed, Treg cells are likely protective in MS. For example, the transfer of myelin oligodendrocyte glycoprotein specific Treg cells displayed dose-dependent protection against experimental autoimmune encephalomyelitis [175, 258]. Inflammation in MS pathology may be in part due to a decrease in Treg cells, [136, 248] or functionality [92, 120, 179, 211, 326]. A reduced capacity of naïve CD4 + T cells to differentiate into Treg cells under pathological conditions has also been demonstrated [281]. A reduction in number or function of Treg cells would mean a decreased capacity for inflammatory suppression. On the other hand, some studies have shown relative increases in the levels of Treg cells in MS [92, 179], but reduced functionality [179]. This may suggest Treg cells functionality may be more consequential in MS pathology than the number itself. Although more research is needed to clarify how each subset of T cells is involved in MS, current data suggest that a failure in Treg cell number and or functioning combined with an upregulation of effector T cells contribute to the inflammation and CNS damage seen in MS [211]. A deeper understanding of T cells interaction within MS will hopefully lead to new therapy. Interventions that downregulate effector T cells or upregulate Treg cells may decrease the disease progression in MS patients.

## Adaptive immunity and the eye

The eye is an “immune privileged” organ, which limits its inflammatory immune response so that vision is not harmed by swelling, infection, and other tissue changes. The eye is similar to the brain, testes, placenta, and fetus in regard to immune responses. Typically, even foreign antigens do not trigger immune responses in these organs. In addition, the blood–retinal barriers in the eye stop infiltration of blood-borne pathogens and immune cells under physiological conditions. However, under pathological conditions such as uveitis, glaucoma, diabetic retinopathy, and retinal ischemia, numerous immune cells can infiltrate the eye and may induce or facilitate autoimmunity that can lead to the development of autoimmunity [38].

In general, if an immune-privileged organ is damaged, previously insulated proteins will be exposed to peripheral immune cells that have not encountered these “novel” antigens. Having never come across these antigens before, peripheral immune cells have not learn to recognize them as self, allowing the generation of autoreactive lymphocytes [99]. The immune-privileged status of the eye is maintained by the blood–retinal barrier’s passive physical sequestration via tight junctions and the retinal pigment epithelium. The protective microenvironment of the eye is immunosuppressive and it expresses substances such as Qa-1, Fas ligand, and indolamine dioxidase (IDO) which function to prevent a damaging inflammatory reaction to ocular tissues [61]. The immunosuppressive environment is also influenced by the aqueous humor which dampens the activity of many immune responses including nitric oxide production by macrophages [310], complement activation [111], neutrophil activation [303], lymphocyte proliferation [162], and NK cell activity [10]. Treg cells are also involved in the immune privilege of the eye through anterior chamber-associated immune deviation [13], in which injection of a foreign antigen into the anterior chamber of the eye causes an antigen-dependent down regulation of delayed-type hypersensitivity [324]. Overall, these mechanisms create an environment that is sheltered from potential immune cell-induced injury [227].

## Adaptive immunity and uveitis

Typically, uveitis is classified by the affected anatomical part of the eye (e.g., anterior uveitis, intermediate uveitis, posterior uveitis and panuveitis) [17, 191]. Often the cause of uveitis is idiopathic, but sometimes an infectious cause (e.g., toxoplasmosis, tuberculosis, onchocerciasis, cysticercosis, leprosy and leptospirosis) can be responsible for this disease [70, 71]. Non-infectious uveitis is immune mediated and can be limited to the eye (e.g., sympathetic ophthalmia and birdshot retinochoroidopathy) or part of a broader systemic disease (e.g., sero-negative HLA-B27-positive spondyloarthropathies, juvenile idiopathic arthritis, sarcoidosis, multiple sclerosis, inflammatory bowel disease, tubulointerstitial nephritis, Behçet disease, and Vogt–Koyanagi–Harada syndrome) [39, 71]. Ongoing research proposes that noninfectious uveitis may be an autoimmune condition through breakdown of self-tolerance and mobilization of autoreactive effector cells. However, there are some theories in the field suggesting some cases of idiopathic immune-mediated uveitis might be the result of reactivatable infectious agents concealed in ocular tissue rather than true autoimmunity [98]. Nevertheless, autoimmune pathogenesis is generally accepted as a contributing factor in uveitis [17]. Support for the autoimmune theory of uveitis is provided by the increased genetic susceptibility of people with certain HLA phenotypes, as HLA genes have long been linked to autoimmunity [205]. In addition, autoimmune conditions are characterized by autoreactive T cells targeting self-antigens, evidence of which has been demonstrated in patients with noninfectious uveitis with uveal melanin, retinal arrestin, and inter-photoreceptor retinoid binding protein (IRBP) [39]. The experimental autoimmune uveitis (EAU) mouse model of posterior uveitis has demonstrated reduced inflammation through anti-CD3-mediated T cell suppression [306]. Interestingly, another study that used an anti-CD3 antibody saw a decrease activation of effector T cells but enhanced activation of Treg cells [168]. The autoreactive T cells responsible for autoimmune pathology were thought to be Th1 CD4 + T cells [80, 100], but more recent studies have indicated both Th1 and Th17 cells can contribute [133, 337]. Most research implicates both Th1 and Th17 in uveitis pathology, but other subsets may also be involved as well, such as a small subset of T cells known as γδ T cells [66, 283]. Some studies have also reported the possibility of autoreactive CD8 + T cells involvement in uveitis [210, 283, 300]. It is also possible that the etiology may vary between the different conditions and the stages of uveitis. For example, in Behçet’s uveitis there is a greater number of CD8 + T cell in the aqueous humor [356], but in sarcoid uveitis the CD4 + /CD8 + ratio was increased [69]. These discrepancies suggest a possible difference in the pathogenesis between uveitis etiologies which could also affect their treatment approaches. There is still a lot to be done to fully clarify the role of CD8 + T cells in uveitis pathology, but there is mounting evidence that CD8 + T cells participate in autoimmune disease, making it plausible that they contribute significantly in uveitis pathology [76, 350]. Despite some remaining ambiguity, there is substantial support that Th1 cells and Th17 cells are implicated in autoimmune pathology [7, 115, 200, 308]. The specific role of each T cell subset in uveitis requires additional research that will fill in the gaps in our knowledge.

Most of the studies have focused on the roles of T cells, but B lymphocytes also likely play roles in uveitis. B lymphocytes may promote an inflammatory environment as well as promote T cell survival [296]. For example, depletion of B lymphocytes by rituximab and other monoclonal antibodies treatment in uveitis provides positive outcomes [68, 128, 214]. In contrast, some studies also have shown protective effects of B lymphocytes. For example, B lymphocytes suppressed intraocular inflammation and helped expand protective Treg cells in a mouse model [55]. Additionally, loss of a transcription factor in B cells caused suppression of both B regulatory and T regulatory cells, which resulted in worsening of the disease [232]. This suggests that certain B cell subsets may be contributing to uveitis pathology, while others may be protective.

Studies have shown that regulatory T (Treg) cells in uveitis have the ability to modulate inflammation and downregulate effector immune functions. The unregulated inflammation seen in uveitis suggests aberrant activation or a breakdown in proper Treg cells functionality or number. This idea was supported by a study done by Muhammad et al. that showed reduced ability to induce Treg cells in the experimental autoimmune uveoretinitis model [220, 333, 334]. In addition, a reduction in Treg cells has been shown in patients with active uveitis [269, 354] and Behçet’s disease before an ocular attack [222] in peripheral blood samples. Dysregulation of Treg cells has also been shown as a contributing factor for disease recurrence in recurrent experimental autoimmune uveitis [167]. Additionally, in a mouse model of experimental autoimmune uveitis adoptive transfer of Treg cells has been shown to suppress disease progression [290]. This idea is further supported by a study that claims a shift away from an inflammatory T cell phenotype in favor of Treg cells help mediate disease resolution [108]. Silver et al. also implicate Treg cells in the resolution and remission of uveitis pathology, although they claim Treg cells functionality is not impaired under pathologic condition [293]. This is a direct contradiction of what Ke et al. state when they claim that dysregulation and improper function of Treg cells contribute to disease reoccurrence [167]. Nevertheless, despite this discrepancy in whether Treg cells are decreased in number only or also in functionality, promoting the proliferation of Treg cells results in the suppression of pathology. These studies implicate Treg cells dysfunction during the development of uveitis. Moreover, a deficiency of Treg cells (e.g., function, numbers) may play a central role in disease pathogenesis. More research is needed to clarify how the population of Treg cells changed during the progression of uveitis.

## Adaptive immunity and diabetic retinopathy

Diabetic retinopathy is one of the leading causes of blindness in human between the ages 27 and 75, and its estimated prevalence is 90% for patients who have had diabetes for over 20 years [41]. Chronic poor glucose control along with diabetes can lead to vascular complications such as macular edema, neovascularization, and microaneurysms which result in the loss of central vision in diabetic retinopathy patients [188]. As the incidence of diabetes continues to rise, the number of people suffering from diabetic retinopathy is expected to rise as well [295]. Many factors contribute to the development of diabetic retinopathy, but this perspective review will provide limited information for the role of T cells in the diabetic retinopathy.

Inflammation has been linked to obesity and metabolic disorders such as diabetes [134, 338]. Many pro-inflammatory cytokines have been shown to be elevated in the vitreous humor of patients with diabetic retinopathy including TNF-α [366], IL-8 and MCP-1 [130], IL-6 [119], IL-26 [333, 334] and IL-1β [366]. Studies have also shown that more Th1-dependent pro-inflammatory cytokines are secreted in diabetic retinopathy, suggesting an imbalance of lymphocytes [36]. An important part of diabetic retinopathy pathology is blood vessel angiogenesis which is promoted by vascular endothelial growth factor (VEGF). However, studies have shown that the Th1/Th2 ratio is an independent predictor of VEGF plasma levels in diabetic retinopathy [363]. Other studies suggest that Th17 cell-dependent IL-17 may be associated with the inflammation observed in diabetic retinopathy [46, 48, 148]. IL-17A has been shown to be an important detrimental cytokine in the progression of diabetic retinopathy [253, 254]. Studies have also shown that Th17 cells can infiltrate the retina in a diabetic retinopathy mouse model [291]. Moreover, elevated levels of IL-17 have been identified in the plasma [124], vitreous [151], and aqueous humor [93], of diabetic retinopathy patients. However, conflicting data exist on the level of IL-17 in the serum of diabetic retinopathy patients because studies have also shown a negative association with IL-17 and diabetic retinopathy [3, 221].

The overwhelming inflammation seen in diabetic retinopathy may be due to an imbalance of Treg cells. For example, studies have shown decreased numbers of Treg cells in type II diabetes and diabetic retinopathy patients [251, 351, 358]. Treg cells have also been shown to be beneficial in repairing abnormal angiogenesis in diabetic retinopathy [74]. In addition to the disturbance in the homeostasis of effector T cells and the population of Treg cells, Treg cells functions could also be altered by the surrounding environment. For instance, Treg cells may be unable to properly suppress inflammation in diabetic retinopathy due to elevated insulin levels [123]. Treg cells have been shown to have reduced suppressive capacity in type II diabetes mellitus patients [285]. Additional studies are required to clearly understand the neuroprotective role of Treg cells in diabetic retinopathy.

## Adaptive immunity and age-related macular degeneration

Age-related macular degeneration (AMD) is a progressive disease-causing degeneration of the macula and is the leading cause of blindness among the elderly population in developed countries [344]. Two different pathologic components contribute to vision loss in AMD: focal atrophy of the retinal pigment epithelium (RPE) and photoreceptor loss (“dry” AMD) or choroidal neovascularization (“wet” AMD) [322]. Typically, dry AMD is a precursor for wet AMD, but not all patients will experience both [8]. In dry AMD, there is a gradual expansion of the atrophic lesions and a slow progressive vision loss. In wet AMD, the new blood vessels leak leading to edema, retinal damage, and can cause a rapid loss of visual acuity. Currently, there is no approved drug therapy to treat dry macular degeneration, though there are many ongoing clinical trials to address this problem [238]. Some clinical progress has been made to treat wet AMD, such as anti-VEGF therapy that targets VEGF, an important growth factor that facilitates angiogenesis [170]. However, anti-VEGF therapy only delays disease progression and upon treatment cessation relapse is a common problem [333, 334]. An important component of AMD is persistent inflammation [239]. Elevated levels of complement proteins have been detected in the blood samples of AMD patients, suggesting some sort of complement dysfunction may be contributing to the disease [126, 202, 263]. The current understanding of AMD is that local complement dysregulation is involved in the disease pathogenesis. One component of complement called complement factor H (CFH) is an important inhibitor of the alternative pathway of complement, and CFH polymorphisms have been linked to an increased risk of AMD development [88, 183]. Despite the abundance of genetic evidence linking complement to AMD, the exact role of how complement may be involved is still not clear.

Studies in an AMD mouse model have shown that T cells can contribute to AMD pathogenesis [65]. Patients with neovascularization AMD were also shown to have higher systemic lymphocyte counts, suggesting that lymphocytes may play an active role in initiating the neovascularization seen in AMD. [305]. Several studies found that the neutrophil-to-lymphocyte ratio (NLR), which are thought to be a marker of both inflammation and angiogenesis, were elevated in wet AMD [147, 225, 309]. These studies suggest that immune cells may be dysfunctional in neovascular AMD, however, the clinical relevance remains unclear [225]. In addition, increased recruitment of T cells in AMD has also been shown [216, 224]. Studies have also shown dysfunction or senescence of the immune system in the context of aging and neurodegenerative disease through the creation of a chronic state of low-grade inflammation referred to as “inflammaging” [73, 101]. This state of immune senescence has been implicated in AMD in which T cells are likely involved [190]. Additionally, several studies have shown an increased proportion of aged T cells in AMD patients, suggesting that immune dysfunction may play a role in disease pathogenesis [89, 305]. Dysregulation of follicular T cells has also been reported in AMD [345]. The idea of T cells dysregulation is further supported by a study showing alterations in systemic Th1 lymphocyte profiles in AMD patients [294]. More specifically, studies have shown that IL-17 may be an important part of AMD disease development [11, 288] since reducing IL-17 levels decreased the amount of choroidal neovascularization [125, 197]. However, unlike other diseases previously discussed in this review, the main source of IL-17 in AMD may not be from Th17 cells, but instead from γδ T cells [365]. These IL-17 producing γδ T cells have been shown to infiltrate the eye in a mouse model of choroidal neovascularization (CNV) [64]. Notably, IL-17 + cells have also been identified near areas of RPE atrophy [35]. Another mouse model study showed that Th2 cytokines, mainly IL-4, helped to decrease neovascularization in the disease process [346]. This supports the overall theory that an imbalance in the pro/anti-inflammatory phenotypes helps to drive AMD. Limited data have shown an increase in Th17 and Th1 cells in AMD patients [49, 50], whereas another study has shown a decrease in Th1 cells and no significant changes in Th17 cells [294]. Interestingly, one study found that decreased levels of CD4 + T cells correlated with the absence of subretinal fibrosis in AMD [187]. Overall, more studies are needed to better understand the role of adaptive immune cells in AMD.

Limited literature exists for the involvement of B lymphocytes in AMD, but anti-retinal antibodies have been identified in AMD patients [2, 118, 146, 217, 243]. It remains in question whether they are a consequence of disease-related damage or a contributory factor for the disease development [157, 158, 160]. In contrast, studies have also shown no difference in the number of circulating B lymphocytes in AMD patients when compared with healthy subjects, which does not rule out B lymphocytes involvement in AMD pathology because antibody production by B lymphocytes could still have a contributory effect [46, 48]. Studies have also suggested that autoantibodies could be used as biomarkers for future disease progression and prognostication [157, 158, 160, 218]. Another study found elevated levels of IL-17 correlated with response to anti-VEGF therapy [223]. Identification of a biomarker could have great clinical significance in AMD treatment, however more work is needed in this area.

Retina repair and reduced angiogenesis were observed through adoptive transfer of Treg cells and Treg cells expansion [74]. Currently, not many studies have looked at the role of Treg cells in AMD. One study did not find changes in the number of systemic Treg cells in AMD [203]. Like effector T cells and B cells, it is again possible that Treg cells dysfunction may play larger roles than Treg cells number [19]. Treg cells are a promising candidate to study in inflammatory diseases, and they may be implicated in AMD, though more research is needed.

## Adaptive immunity and glaucoma

Glaucoma is the second leading cause of blindness worldwide in which retinal ganglion cells (RGCs) die slowly and progressively over a prolonged period of time. Glaucoma is now considered to be a multifactorial disease and molecular mechanisms involved in RGC death are complex and poorly understood [28, 109, 226, 325]. The primary risk factor for developing glaucoma is elevated intraocular pressure (IOP), but the pathophysiology of the disease is more complicated [22, 59, 84, 127, 299]. There is currently no known cure for glaucoma and treatment focuses on reducing IOP [47]. Unfortunately, some patients under IOP-lowering therapy still see progression of the disease, which clearly indicates that better therapeutic options are needed to fully cure the disease. Studies have shown that multiple factors play key roles in RGC degeneration including activation of caspases [44, 141, 142, 169, 208, 320], apoptosis [6, 116, 226, 255, 327], oxidative stress [56, 143, 150, 198, 219, 228, 272], ischemia and hypoxia [32, 57, 141, 141, 142, 142, 144, 234], epigenetic changes [105, 206, 242, 276, 353, 359, 359, 360, 360], Crosson 2010 #975, [145], alteration in the levels of pro-inflammatory cytokines [1, 312, 316, 341, 352, 357], and deprivation of neurotrophic factors [240, 256]. There is another opinion with limited evidence, which suggests the neurodegeneration observed in glaucoma could be vascular. This theory suggests that hemodynamic alterations and changes in local blood flow may cause unstable ocular perfusion to the optic nerve [42, 54, 96, 237, 355]. Recently, studies have also shown participation of T cells in glaucoma pathology. A study has shown that elevated IOP allowed for T cell infiltration of the retina and led to continued degeneration of RGCs after IOP was returned to a normal level [45, 51].

The innate immune system has long been tied to glaucoma through the action of glial cells and oxidative stress [201, 314, 319], but recent evidence provides additional support for the involvement of the adaptive immune system in glaucoma pathology [156]. Earlier studies have shown that glaucoma could be critically tied to the immune system because resistance of RGC death was shown to be correlated with immune potency [16]. This study suggested that immune dysfunction may be a prerequisite for developing glaucoma and would explain the disparities in degree of disease progression among patients treated with ocular hypertensive agents. Afterwards, numerous studies have shown the presence of autoantibodies to retinal and optic nerve proteins in the serum [21, 77, 159, 204, 264, 313, 317, 348], retina [112], and aqueous humor [157, 158, 160] of glaucoma patients. Studies have also shown the presence of autoantibodies to heat shock proteins [26, 107, 318, 323, 336] in glaucoma. Heat shock proteins are a large family of molecular chaperones that can be upregulated in times of stress [213], but also have significant potential to induce molecular mimicry resulting in the creation of autoantibodies to host proteins [335]. In glaucoma, elevated levels of heat shock proteins such as HSP72 [318], HSP60, and HSP27 [315], have been reported, some of which may help facilitate RGC death [318, 330]. Introduction of heat shock proteins to rats through immunization can induce glaucomatous injury [45, 51, 336], but other studies have shown that induction of heat shock proteins can provide neuroprotection for RGCs [37, 236, 252]. Overall, the role of autoantibodies and heat shock proteins in glaucoma remains unclear. It is not clear if they are involved in glaucoma pathology directly, indirectly, or play a role in protective autoimmunity [364].

The involvement of T cells in glaucoma pathology remains in question, but we believe that T cell subsets, specifically the T effector/Treg cells homoeostasis, play a critical role in determining the fate of RGCs during glaucoma progression. Additionally, studies have shown an adoptive transfer of lymphocytes from glaucomatous mice into healthy mice stimulated RGC loss [113]. This causative effect suggests that adaptive immune dysfunction plays a direct role in the pathophysiology of glaucoma. Studies have shown that CD4 + T cells facilitate RGC death in an acute ischemia/reperfusion injury model [137]. An imbalance in Treg cells/Th17 cells in experimental autoimmune optic neuritis (EAON) has been shown, which shares key characteristics of glaucoma pathology [199]. Recent studies have also shown that transient elevation of IOP can cause T cells infiltration to the retina, leading to RGC degeneration [45, 51]. However, the role of Treg cells in glaucoma remains fully unexplored. The authors’ perspective in this regard is “enhanced neuroinflammation during glaucoma could be due to low number and/or function of Treg cells”. In other systems, unchecked inflammation has been attributed due to improper functioning of Treg cells [343]. Adoptive transfer of Treg cells has shown promise in treating inflammatory conditions such as enteritis and multiple sclerosis suggesting it may prove an effective therapeutic option in glaucoma [87]. If under glaucomatous condition Treg cells are unable to differentiate, adoptive transfer of Treg cells may ameliorate the T cell imbalance and decrease inflammation. More work is needed in this field to better understand the involvement of T cells in glaucoma progression.

## Conclusions

**Fig. 1:**
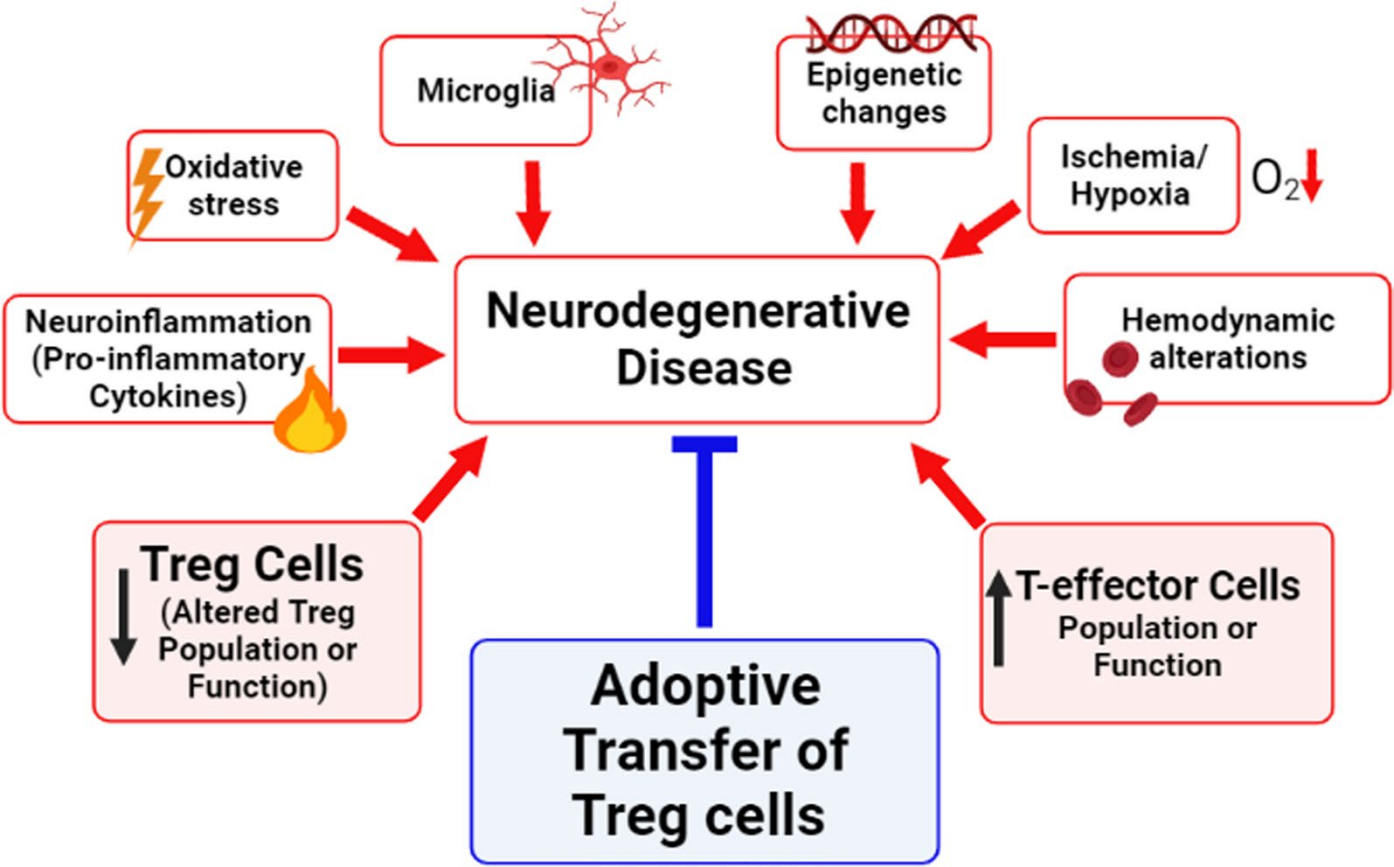
Schematic showing the roles of numerous factors and immune cells in the induction or protection in neurodegenerative diseases

We provided a brief overview of adaptive immunity, autoimmunity, and tolerance and related them to neurodegenerative and ocular diseases. This perspective review article aims to emphasize the significance of adaptive immunity concerning neurodegenerative conditions and highlight the gaps in the field. It also highlights the pathological and neuroprotective roles of different subsets of lymphocytes in numerous neurodegenerative diseases, including Alzheimer’s disease, Parkinson’s disease, multiple sclerosis, and several ocular diseases. Figure 1 provides a framework of potential contributing factors to neurodegenerative diseases. Although the etiology of each neurodegenerative disease is different and complex, a few common players that may have crucial roles in the pathology have been shown. These factors include neuroinflammation, epigenetic changes, ischemia/hypoxia, oxidative stress, hemodynamic alterations, and changes in the immune cells. The focus of this perspective review is to provide information for the role of immune cells (e.g., effector T and Treg cells) in neurodegenerative diseases. Based on the literature in non-ocular and ocular neurodegenerative diseases, we provided our perspective that: (1) the number of effector T cells may be increased under ocular pathologies such as uveitis, diabetic retinopathy, AMD, and glaucoma. Elevated T cells can subsequently enhance the secretion of pro-inflammatory cytokines and expedite retinal degeneration similar to other neurodegenerative diseases, and (2) Treg cells are deficient in function and/or number rendering them unable to sufficiently regulate the function of effector T cells in ocular pathologies as seen in other neurodegenerative diseases. Evidence of lymphocyte involvement in ocular pathologies is promising and there is a vital need for more research in this field. We conclude that T cell subsets homeostasis is critical for the maintenance and regulation of neuroinflammation. If the homeostasis is lost and the balance of specialized subsets of immune cells breaks down, damage to the host’s tissues can lead to pathological conditions. The inability to yet discover a neuroprotective therapy for ocular pathologies (e.g., uveitis, diabetic retinopathy, AMD, and glaucoma) it is highly desirable to target immune cells for future research.

Based on the literature provided in this perspective review article, we believe that in ocular neurodegenerative diseases, there may be an imbalance in the number of effector T cells as well as the number and or function of Treg cells, which might contribute to a pro-inflammatory state and facilitate neuronal death in such ocular diseases (e.g., uveitis, diabetic retinopathy, AMD, and glaucoma). This area of research is underdeveloped, hence more studies are needed to clearly understand how T cell subsets and dysfunction may play a role in developing ocular neurodegenerative diseases.

## Data Availability

Not applicable.

## Abbreviations

AMD: Age-related macular degeneration
APC: Antigen presenting cell
CFH: Complement factor h
CNS: Central nervous system
EAE: Experimental autoimmune encephalomyelitis
EAON: Experimental autoimmune optic neuritis
EAU: Experimental autoimmune uveitis
HLA: Human leukocyte antigen
HSP: Heat shock protein
IDO: Indolamine dioxidase
IFN-γ: Interferon gamma
IL-1β: Interleukin-1-beta
IL-4: Interleukin-4
IL-5: Interleukin-5
IL-6: Interleukin-6
IL-8: Interleukin-8
IL-10: Interleukin-10
IL-13: Interleukin-13
IL-17: Interleukin-17
IL26: Interleukin-26
IOP: Intraocular pressure
IRBP: Inter-photoreceptor retinoid binding protein
MCP-1: Monocyte chemoattractant protein-1
MHC: Major histocompatibility complex
MS: Multiple sclerosis
NADPH: Nicotinamide adenine dinucleotide phosphate
NK cells: Natural killer cells
PD: Parkinson’s disease
RGC: Retinal ganglion cells
TNF-α: Tumor necrosis factor-alpha
TNF-β: Tumor necrosis factor-beta
VEGF: Vascular endothelial growth factor
WBCs: White blood cells
